# Zebrafish Her8a Is Activated by Su(H)-Dependent Notch Signaling and Is Essential for the Inhibition of Neurogenesis

**DOI:** 10.1371/journal.pone.0019394

**Published:** 2011-04-26

**Authors:** Pei-Chen Chung, Wen-Shiuan Lin, Paul J. Scotting, Fu-Yu Hsieh, Hui-Lan Wu, Yi-Chuan Cheng

**Affiliations:** 1 School of Medicine, Graduate Institute of Biomedical Sciences, Chang-Gung University, Taoyuan, Taiwan; 2 Children's Brain Tumour Research Centre, Centre for Genetics and Genomics, Queen's Medical Centre, University of Nottingham, Nottingham, United Kingdom; Instituto de Medicina Molecular, Portugal

## Abstract

Understanding how diversity of neural cells is generated is one of the main tasks of developmental biology. The Hairy/E(spl) family members are potential targets of Notch signaling, which has been shown to be fundamental to neural cell maintenance, cell fate decisions, and compartment boundary formation. However, their response to Notch signaling and their roles in neurogenesis are still not fully understood. In the present study, we isolated a zebrafish homologue of *hairy/E(spl)*, *her8a*, and showed this gene is specifically expressed in the developing nervous system. *her8a* is positively regulated by Su(H)-dependent Notch signaling as revealed by a Notch-defective mutant and injection of variants of the Notch intracellular regulator, Su(H). Morpholino knockdown of Her8a resulted in upregulation of proneural and post-mitotic neuronal markers, indicating that Her8a is essential for the inhibition of neurogenesis. In addition, markers for glial precursors and mature glial cells were down-regulated in Her8a morphants, suggesting Her8a is required for gliogenesis. The role of Her8a and its response to Notch signaling is thus similar to mammalian HES1, however this is the converse of what is seen for the more closely related mammalian family member, HES6. This study not only provides further understanding of how the fundamental signaling pathway, Notch signaling, and its downstream genes mediate neural development and differentiation, but also reveals evolutionary diversity in the role of *H/E(spl)* genes.

## Introduction

The nervous system consists of a diverse collection of neural cells that arise during a sequence of developmental events including neural induction, cell proliferation, differentiation, migration, process formation, and synapse formation. These events require numerous gene regulatory and signaling processes. One of the key regulators in these processes is Notch signaling which is conserved throughout evolution and components of this pathway have been characterized both in *Drosophila* and vertebrates [1]. In the developing nervous system, Notch signaling plays fundamental roles in neuronal progenitor maintenance and the decision between neuronal and glial lineages. It also influences aspects of the behavior of terminally differentiated neurons and is important in patterning cellular fields during brain morphogenesis. In addition, Notch signaling also participates in the pathogenesis of several human cancers and genetic disorders [2].

The Notch receptor is a single transmembrane protein that acts as both a sensor for ligand-activation and a mediator of the resulting signal to the cell nucleus. Notch signaling regulates neural fate commitment upon interaction with its canonical ligands Delta or Serrate (JAGGED in mammals). These trigger proteolytic cleavages that release the Notch intracellular domain (NICD) to enter the nucleus. NICD then interacts with the DNA binding protein CSL [CBF/RBP-J, Su(H), LAG-1], leading to the transcription of target genes, *Hairy and Enhancer-of-split* [*Hairy/E(spl)*]. Hairy/E(spl) related proteins are transcription factors belong to the bHLH superfamily and are structurally related to the *Drosophila* Hairy and Enhancer-of-split proteins. It is generally believed that Hairy/E(spl) homologues act as potential targets for Notch mediated signals that suppress the expression of proneural genes such as *Neurogenin1* and *Achaete-scute homolog 1*, resulting in inhibition of neurogenesis [1]. However, some *Hairy/E(spl)* genes have been identified that do not respond to Notch signaling ([3], and reference therein) and a recent study showed that a zebrafish *hairy/E(spl)* homologue, *her3*, is repressed rather than induced by Notch signaling [4]. It therefore appears that not all the *Hairy/E(spl)* genes respond to Notch signaling in the same way, and the regulation of these genes' expression may vary in different developmental stages and different cell populations. In addition to the variable response to Notch signaling, the function of Hairy/E(spl) proteins themselves is also controversial. Most of the Hairy/E(spl) homologues studied so far act as transcriptional repressors for the proneural bHLH genes, and thus inhibit neurogenesis. However, one of the mouse homologues, HES6, has been shown to promote rather than inhibit neurogenesis [5], [6]. Hence, our understanding of the roles of Hairy/E(spl) in neurogenesis and their response to Notch signaling remains incomplete.

The zebrafish, *Danio rerio,* has emerged as an excellent vertebrate model, with advantages over other organisms for the study of many aspects of developmental biology. In particular, the morpholino approach provides efficient and economic phenocopies of gene mutations. To date, more than 20 zebrafish Hairy/E(spl) homologues, named Her [zebrafish homologue of Hairy/E(spl) related] and Hey [Hairy/E(spl) related with YxxW motifs], have been identified [7]. Nine genes have been reported to exhibit expression in the developing nervous system (*her3*: [4]; *her4*: [8]; *her5*: [9]; *her6*: [10]; *her9*: [11]; *her11*: [7]; *her12* and *her15*:[7]; *hey2*: [12]) and six (*her3*, *her4*, *her5*, *her6*, *her9*, *her11*) have been functionally analyzed. Among those, Her3, Her4, Her5, Her9 and Her11 have been shown to repress proneural or neuronal genes, and expression of *her3* and *her5* is repressed by Notch activation whereas *her4* and *her6* are induced by Notch signaling, and *her9* is independent of Notch activation ([4], [9], [13], [14], [15]). Outside of the nervous system, some of the *her* genes are also expressed in the presomitic mesoderm and are essential for somite segmentation [3].

Here we present the isolation of a previously uncharacterized Hairy/E(Spl) homologue, *her8a*, in zebrafish and show that its expression is restricted in the developing nervous system. *her8a* can be grouped into the *Hes6* subgroup by sequence similarity. However, unlike mammalian *Hes6*, *her8a* is regulated by Su(H)-dependent Notch signaling and it is essential for the inhibition of neurogenesis. We also found Her8a to be required for gliogenesis. Our data provide further insights into the diverse roles of *Hairy/E(Spl)* homologues in neural development and their response to Notch signaling.

## Results

### Characterization of zebrafish *her8a*

**Figure 1:**
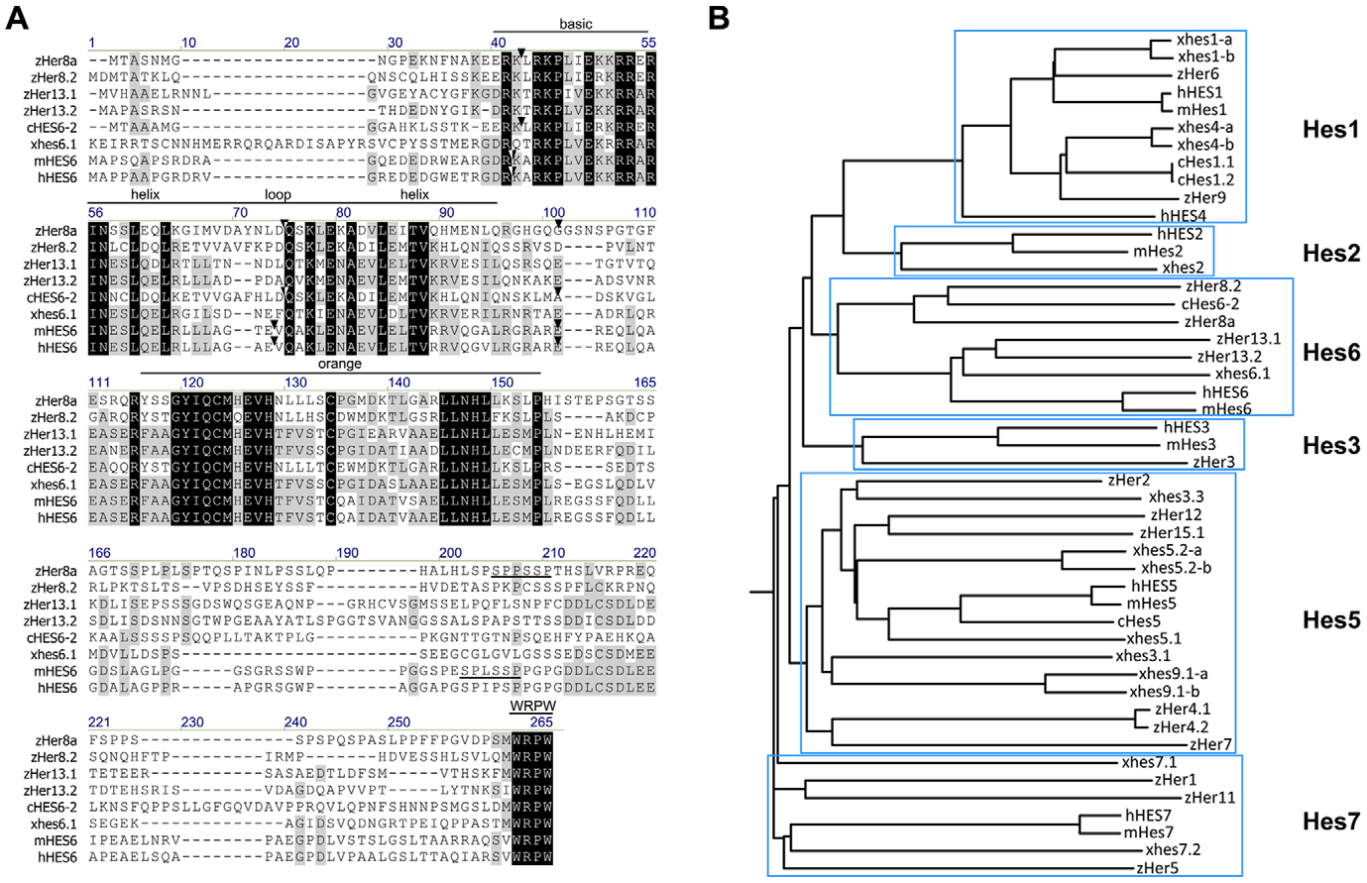
Alignment and phylogenetic analysis of Her8a and the H/E(spl) family. (A) Amino acid alignment of human, mouse, Chicken, *Xenopus laevis*, and zebrafish Hes6 related sequences. Residues identical in all proteins are marked in black boxes and partial conservation is shown in grey boxes. The basic helix-loop-helix domain, orange domain and C terminal WRPW motif are indicated, and LNHLL and SPXXSP motifs are underlined. (B) Phylogenetic tree for the H/E(spl) protein family. The full coding protein sequence was used for each family member. Trees were calculated with a bootstrap support of 100 replicates. The phylogram demonstrates only sequence relationships, but does not absolutely imply sequence ancestry since no ancestral relationship is assumed in initial alignments. The initial letter “h” denotes human, “m” mouse, “c” chicken, “x” *Xenopus laevis* and “z” zebrafish.

Primers were designed according to the zebrafish genome database (www.ensembl.org, version Zv7) to amplify the open reading frame of *her8a* (also known as *her8.1* in [7] and [16]) (GenBank accession number AY007990 and NM_199624). The product of the polymerase chain reaction comprised 663 bp encoding a 221 residue peptide identical to *her8a*. Alignment with the genomic sequence showed the open reading frame was contained within four exons which correspond to exon 1-4 in comparison to the prediction of the Ensembl database (reference number ENSDARG00000016363). The amino acid sequence of Her8a shows structural features of H/E(spl) family members that contains a bHLH domain, a two-helix orange domain and a C-terminal WRPW motif, implying that it may act as a transcriptional repressor (Fig. 1A). Her8a can be further grouped into E(spl) subfamily due to lack of the HC domain present in other Hairy subfamilies [17]. The most similar paralogues of the zebrafish h/E(spl) subfamily are Her8.2, Her13.1.and Her13.2 which show 43%, 32% and 35% identity to Her8a, respectively. Compared to other orthologues of the E(spl) family, Her8a showed the highest degree of similarity to chicken HES6-2 with 50% identical and 66% conserved amino acids [18]and slightly weaker homology with *Xenopus* hes6.1 (previously named hes6 in [6]; 34% identical and 54% conserved amino acids) and mouse HES6 (34% identical and 52% conserved amino acids) (Fig. 1). Within the bHLH domain the identity increases to 71% for chicken HES6-2, 55% for *Xenopus* hes6.1 and 54% for mouse HES6. The three introns interrupt the Her8a protein coding sequence in an identical position in chicken HES6-2 and in a very similar position in mammalian homologues (Fig. 1A). The conservation of intron/exon structure within the *Hes6* genes is strong evidence for evolution from a common ancestor gene specific to this subgroup. In comparison to the well-characterized sequence of mouse HES6, Her8a does not contain the negatively charged acidic residues which are responsible for heterodimerization [19], but it contains an identical LNHLL motif which was shown to be important for inhibition of astrocyte differentiation [20] (Fig. 1A). Conserved SPXXSP motifs were also found at the C-terminus of HES6 and Her8a which are putative MAPK phosphorylation sites [19].

### *her8a* is expressed in the developing nervous system

**Figure 2:**
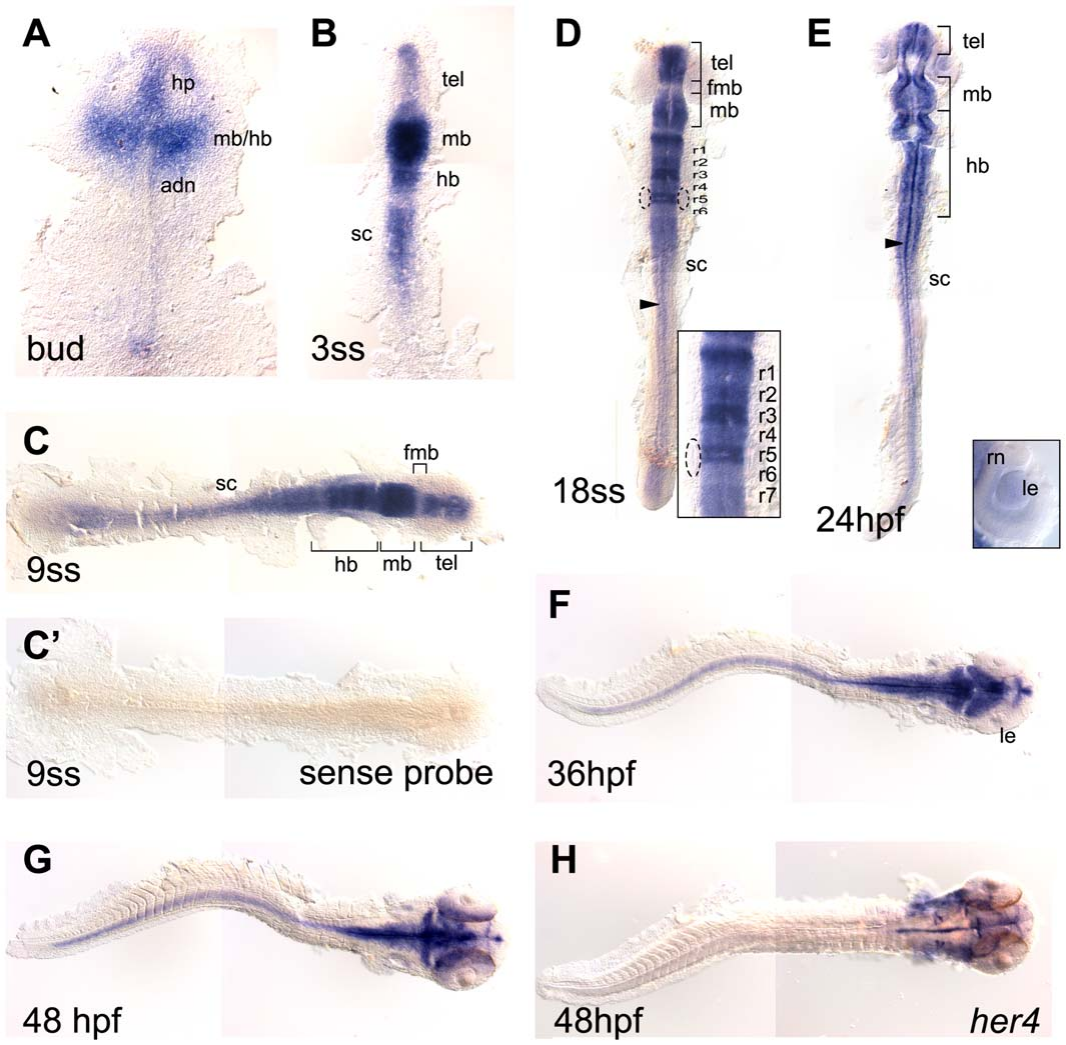
*her8a* expression in the developing zebrafish embryos. *her8a* expression is restricted in the developing nervous system during zebrafish embryogenesis analyzed by *in situ* hybridization. Stages of embryos shown in bottom left corner of each panel. Yolks were removed and embryos were flat-mounted, dorsal view except F and G lateral. *her8a* expression first appears in the developing brain at bud stage (A) and later becomes restricted to specific brain areas (B–E). The transcripts can be detected in the spinal cord from the 3-somite stage (3ss) (B) and retained until the latest stage analyzed (48 hpf) (C–G). Dashed circles in D mark the otic vesicles. Insert panel in D is an enlargement of the hindbrain, and in E is an enlargement of the eye. Arrowheads in D and E indicate *her8a* expressing cells at the midline. No signal was detected using sense riboprobe (C'). (H) *her4* expression at 48 hpf . fmb, forebrain–midbrain boundary; hb, hindbrain; hp, hypothalamus; le, lens; mb, midbrain; r1-r7, rhombomere 1–7; rn, retinal neuroepithelial cells; sc, spinal cord; tel, telencephalon.

*her8a* expression was analyzed by whole mount *in situ* hybridization. Transcripts first appeared in the developing nervous system at bud stage in the primodia of telencephalon, hypothalamus, midbrain and hindbrain (Fig. 2A). From the 3-somite stage onwards, strong *her8a* expression was retained in the telencephalon, midbrain, hindbrain, and started to be expressed in the spinal cord (Fig. 2B–G). The expression in the spinal cord was retained until the latest stage analyzed (48 hpf, Fig. 2F–G). Clear gaps of weak expression could be found in the forebrain–midbrain boundary from the 9-somite stage to 24 hpf (Fig. 2C–E). It was notable that dynamic expression was observed in the developing hindbrain with all rhombomeres expressing *her8a* at different time points and at different levels. During hindbrain segmentation, *her8a* transcripts were initially detected broadly in all rhombomeres (3-somite stage, Fig. 2B), became clearly segmented at 9-somite stage (Fig. 2C) and was later strongly expressed in rhombomere 3, 5, and 7, and comparatively weaker in the other hindbrain segments (Fig. 2D), suggesting Her8a may play a role in hindbrain patterning. This segmental expression pattern gradually disappeared after 24 hpf, instead the expression became restricted to the midline and hindbrain neurons (Fig. 2E). In conjunction with previous studies showing that Her4 and Her9 regulate the specification of midline precursor cells [21], [22], our data suggest that Her8a may also be involved in midline formation. In the developing eyes, transient expression in the lens and the adjacent ventral retinal neuroepithelial cells was detected at 24 hpf but lost by 36 hpf (Fig. 2E). During these stages, the lens epithelium proliferates and differentiates into crystalline fiber cells [23], and the first postmitotic retinal cells appear forming ganglion cells in the ventral retina [24]. Therefore, Her8a may also play a crucial role in retinal neuron proliferation and differentiation. In general, the dynamic expression of *her8a* in neural tissues suggests its importance in central nervous system development.

The expression of *her8a* was also compared to all identified *notch* homologues (*notch1a*, *notch1b*, *notch2* and *notch3*) (Fig. S1). The results showed *her8a* expression most resembled that of *notch1a*, for which significant expression was detected in the brain and anterior spinal cord and also exhibited a segmental pattern in the developing hindbrain from the 9-somite stage (Fig. 2; Fig. S1C1 and D1). A region of weak expression of both genes was also observed in the forebrain–midbrain boundary (Fig. 2; Fig. S1C1 and D1). Nonetheless, *notch1a* was expressed strongly in the posterior spinal cord and tail bud from bud to 9-somite stage where no *her8a* expression could be detected. *notch1b* and *notch2* were ubiquitously expressed in the developing central nervous system, and *notch3* expression was predominantly in tissues outside of nervous system sharing little in common with *her8a* expression (Fig. S1). The results suggest that *her8a* is most likely to be regulated by Notch1a.

*her8a* expression was next compared to those *her* genes which are known Notch targets (*her3* and *her4*) as well as the well-established target of notch signaling, *neurogenin1*, in the developing nervous system (Fig. S1). During early stages of neurogenesis, *her3* was expressed in the inter-proneuronal domains where it represses proneural genes (Fig. S1) [13], [4] and Her3 also negatively regulates *neurogenin1* in rhombomeres 2 and 4 [4]. In comparison, the expression of *her4* was similar to *neurogenin1*, expressed in the proneuronal domains complementary to the inter-proneuronal regions (Fig. S1) [8]. A previous study showed that *her4* can be induced by Notch activation to suppress *neurogenin1* in the proneuronal domains [8]. We found the expression of *her8a* in the spinal cord was more restricted in the middle region which did not resemble *her3* expression but partially overlapped with expression of *her4*. In addition, strong *her8a* expression in the telecephalon was more similar to *her4* expression where no *her3* transcript could be detected (Fig. 2; Fig. S1).

### *her8a* is regulated by the canonical Notch signaling pathway

**Figure 3:**
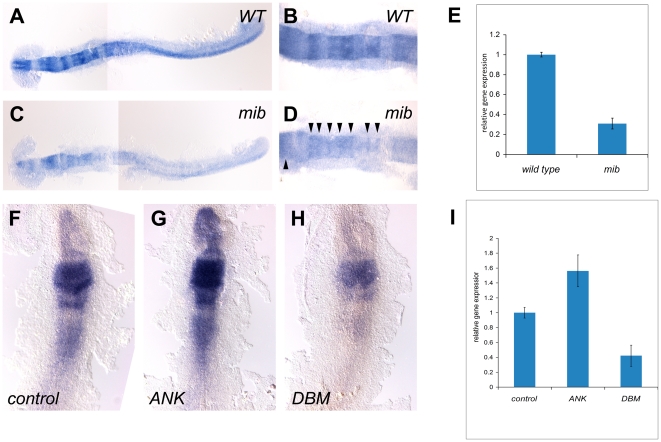
*her8a* is regulated by the Su(H)-dependent Notch signaling pathway. *her8a* is expressed at lower levels in the Notch activity-deficient *mind bomb* mutant embryos (C and D) in comparison to wild-type siblings (A and B). B and D are higher power views of hindbrain regions in A and C, respectively. Note the remnants of *her8a* transcripts in *mind bomb* mutants at the midbrain and hindbrain rhombomere boundaries (arrowheads in D). (E) Quantification of *her8a* expression analyzed by qPCR showing significantly reduced level of *her8a* in *mind bomb* mutants. (F–H) RNA encoding constitutively-active Su(H) (ANK) (G) or dominant-negative Su(H) (DBM) (H) was injected at the one or two-cell stage and analyzed at the 3-somite stage. (F) Control embryos injected with *GFP* mRNA; (G) *her8a* expression is upregulated by constitutive-active Su(H) injection; (H) *her8a* expression is downregulated in dominant-negative Su(H) injected embryos. (I) The levels of *her8a* expression in *Su(H)* variant injected embryos were further quantified by qPCR, showing significantly increased *her8a* expression in *ANK* injected embryos and decreased expression in *DBM* over-expressed embryos. ANK, constitutive-active Su(H); DBM, dominant-negative Su(H), mib: *mind bomb* mutants.

In the developing nervous system, *Hairy/E(Spl)* homologues can suppress or promote neurogenesis acting in either a Notch-dependent or independent manner (reviewed in [25] and [3]). To examine the response of *her8a* to Notch signaling, we analyzed its expression in *mind bomb* mutant embryos (*mib^ta52b^*) that have a strong Notch pathway deficiency [26] due to mutation of a ubiquitin ligase required for Delta ligand activity [27]. We found that in *mib^ta52b^* embryos, *her8a* was significantly downregulated compared to wild-type embryos (100%, N = 30; Fig. 3A–E), suggesting that *her8a* is activated by Notch signaling. Nonetheless, low level expression could be detected in the midbrain and hindbrain boundaries which may due to residual Notch activity in the *mib^ta52b^* mutants (Fig. 3A–D). An alternative explanation is that *her8b* is not dependent upon Notch and could be activated by other signaling pathways in these regions (see Discussion).

Notch signaling can be mediated via Su(H)-dependent (canonical) and Su(H)-independent (non-canonical) pathways ([28]. A putative Su(H) binding sequence GTGGGAA [16] was identified at positon −203 to −197 upstream of the *her8a* translation start codon. To test whether *her8a* is activated through the Su(H)-dependent Notch pathway, we expressed RNAs encoding a constitutive-active form (ANK) or dominant-negative form of Su(H) (DBM) which have been shown to activate or suppress Notch signaling target genes, respectively [29]. The *Su(H)* variants were injected at the 1–2 blastomere stage and harvested at the 3-somite stage for *in situ* hybridization with a *her8a* riboprobe (Fig. 3F–H). The results showed *her8a* transcripts were upregulated in constitutive-active Su(H) injected embryos (70%, N = 50; Fig. 3F) and downregulated after dominant-negative Su(H) injection (80%, N = 55; Fig. 3H), indicating that *her8a* is activated by Su(H)-dependent Notch signaling.

### Loss of Her8a is sufficient to cause a neurogenic phenotype

**Figure 4:**
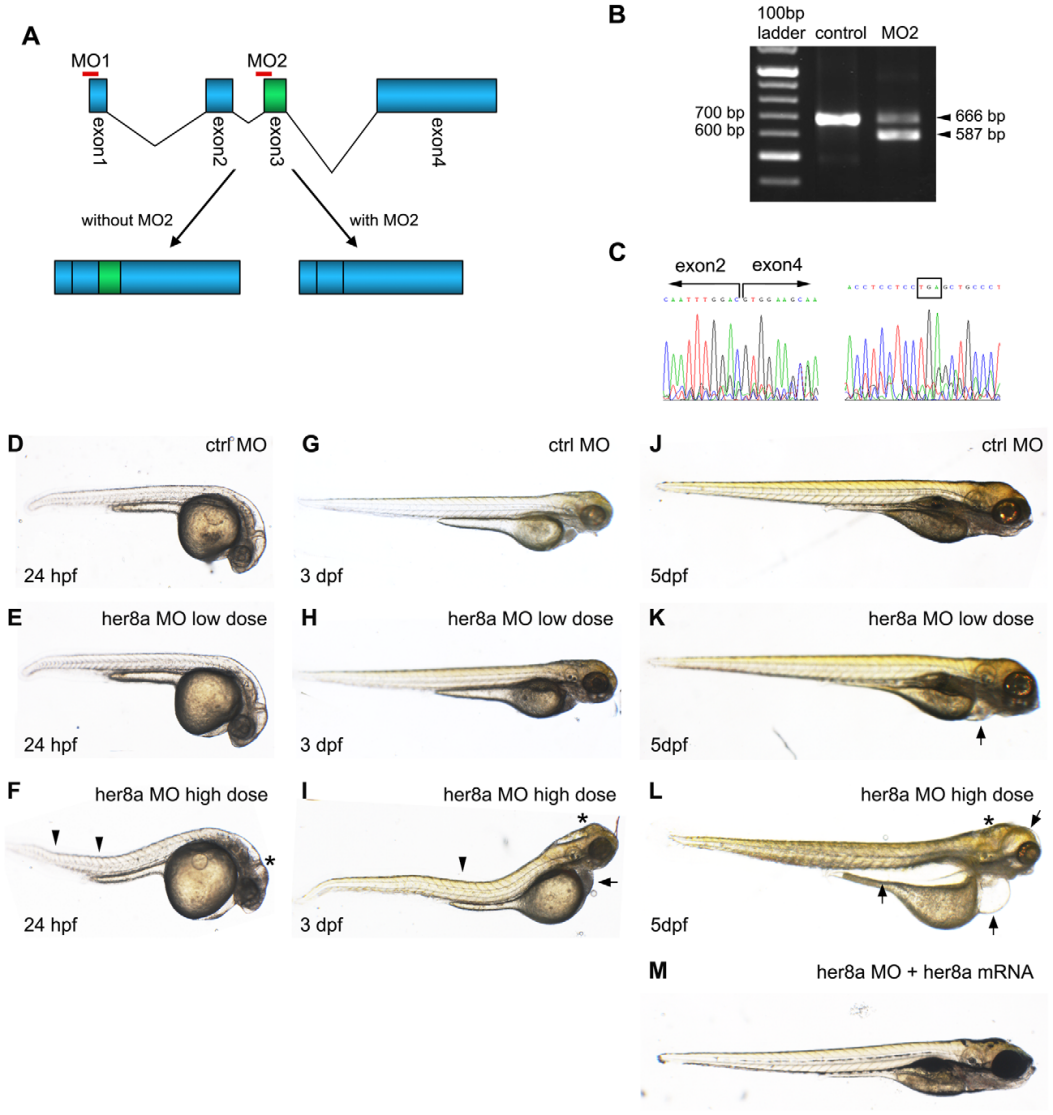
Knockdown of Her8a by specific morpholino antisense oligonucleotides causes developmental abnormalities. (A) Schematic representation shows the genomic organization of the *her8a* gene. Regions targeted by translational-blocking (MO1) and splice-blocking (MO2) morpholinos are shown. (B) The efficacy of MO2 was validated by RT-PCR using primers as indicated in A. Wild-type *her8a* mRNA produces a 666 bp PCR product while alternatively spliced transcripts from morphant embryos yield a 587 bp fragment. (C) The mis-spliced event resulted in loss of exon 3 confirmed by sequencing the 587 bp PCR product in B (left panel). The mis-splicing event resulted in a premature stop codon in exon 4 (boxed, right panel). (D–M) Representative images of morpholino injected embryos. (D, G and J) Control morpholino; (E, H and K) ‘low dose’ indicates 6 ng of MO1 or 4 ng of MO2 injection, resulting in indistinguishable phonotype; (F, I and L) ‘high dose’ represents 12 ng MO1 or 8 ng of MO2 injection, which resulted in identical morphological defects. Arrows denote bent body characteristics, and arrowheads indicate edemas in eyes, pericardial sac and abdominal cavity. Malformation of brains is marked by asterisks. (M) These morphological phenotypes can be rescued by concomitant injection of 2 ng *her8a* mRNA.

To delineate the role of Her8a in neurodevelopment, the morpholino (MO) knockdown approach was used to interfere with its expression. An antisense morpholino (MO1) was synthesized to target the translation start site of *her8a* mRNA to block protein production. Comparison to available database sequences predicted binding of MO1 to *her8a* with no other similar sequence in the zebrafish genome. In order to confirm the specifity of MO1, a second morpholino (MO2) was designed that targets the intron2-exon3 boundary resulting in a truncated product (Fig. 4A). Comparison of MO2 sequence with available zebrafish genomic sequences again predicted that MO2 would specifically binds only to *her8a*. Four other regions with lower identity to MO2 were found, which show less than 20 bp identity compare to the 25 bp MO2 sequence, and these fragments do not correspond to any 5′ UTR or exon-intron splicing site of predicted or characterized genes, suggesting that MO2 would also act specifically on *her8a*. RT-PCR with primers that spanned the MO2 binding sequence was employed and a fragment corresponding to an mRNA lacking the 79 bp of exon 3 was detected in the morpholino injected embryo extract (Fig. 4B). The mis-spliced product was subjected for sequencing, which confirmed exon 3 deletion and further showed a predicted premature stop codon in exon 4 that would severely truncate the encoded protein (Fig. 4C).

Embryos injected with MO1 or MO2 were analyzed at 24 hpf, 3 days post fertilization (dpf) and 5 dpf for morphological defects. 6 ng of MO1 or 4 ng of MO2 injection caused an identical phenotype exhibiting pericardial edema at 5 dpf (Fig. 4K; Fig. S2). In comparison, higher dosage (12 ng MO1 or 8 ng of MO2) injection resulted in more severe defects including brain malformation and a bent trunk from 24 hpf (Fig. 4D–L) and edemas in eyes, pericardial sac and the abdominal cavity from 3 dpf (Fig. 4G–L; Fig. S2). The phenotypes caused by morpholino injection could be rescued by concomitant injection of *her8a* mRNA, indicating that the morpholino-induced defect was due to loss of Her8a function (92%, N = 39; Fig. 4M).

**Figure 5:**
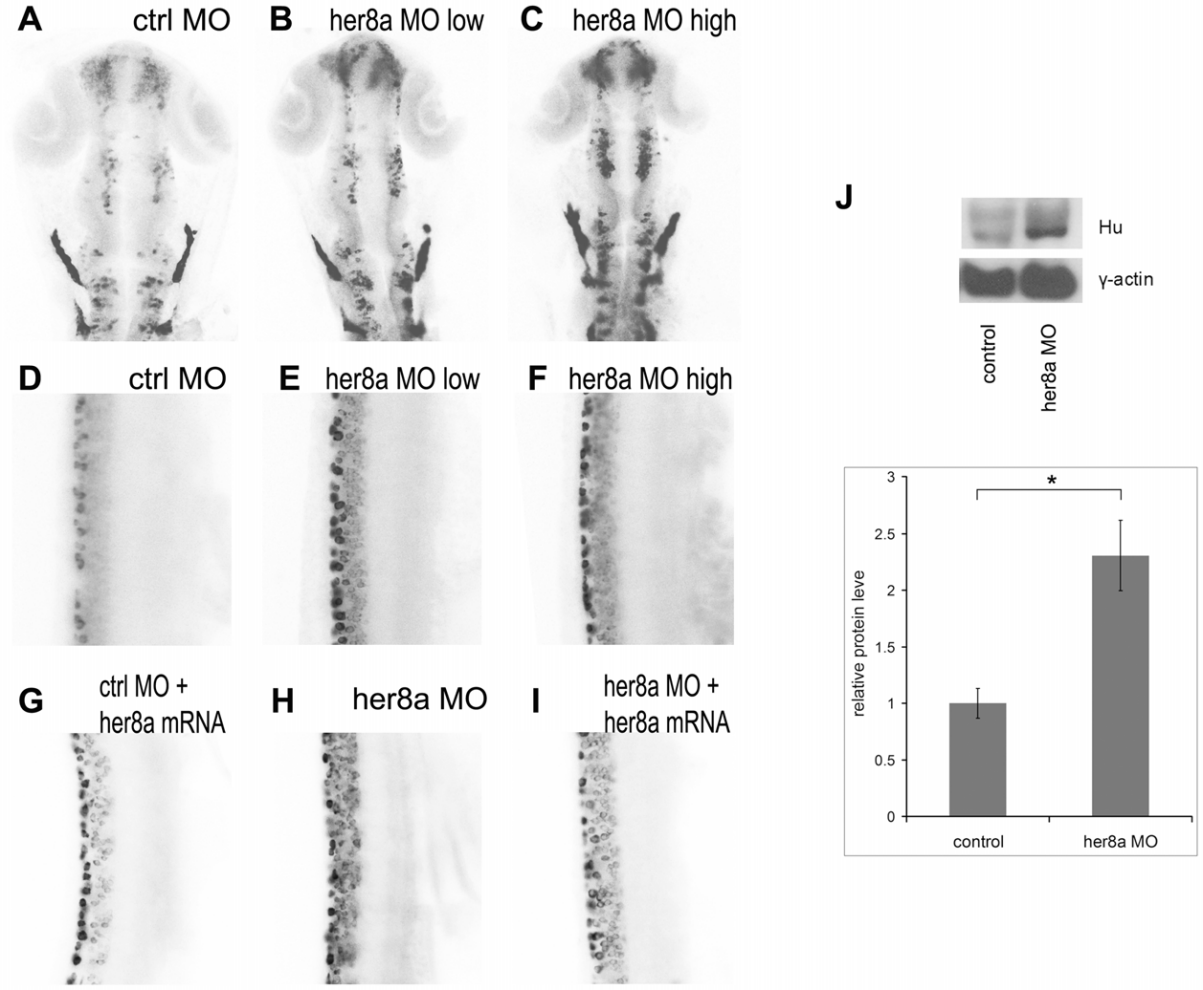
Her8a morphants exhibit upregulation of HuC/D expression. HuC/D expression is upregulated in Her8a morpholino injected embryos analyzed by immunohistochemistry with anti-Hu antibody at 24 hpf. The black-and-white fluorescent signals were inverted to negative film for a better presentation. (A and D) embryos injected with control morpholino. (B and E) 6 ng of MO1 or 4 ng of MO2 injection (low dose) resulted in indistinguishable phonotype and therefore only embryos injected with 4 ng of MO2 are shown (see Fig. S2 for MO1). (C and F) Injection of 12 ng MO1 or 8 ng MO2 (high dose) resulted in identical morphological defects and only MO2 injected embryos are shown, which shows more dramatic upregulation of HuC/D expression. (A–C) Brain regions; (D-I) 3-somite to 9-somite level of the spinal cord. (A–F) Hu-positve cells were significantly increased in the morpholino injected embryos. (G–I) The phenotype can be rescued by co-injection of morpholino with *her8a* mRNA. (J) Western blot analysis confirming the levels of HuC/D expression in Her8a morphants were up-regulated in comparison to the control. * p<0.05.

Many of the Hairy/E(Spl) proteins act as transcriptional repressors to inhibit genes responsible for neurogenesis. In order to address the role of Her8a during neurodevelopment, we analyzed the effect of Her8a knockdown using post-mitotic neuronal marker, HuC/D. Embryos injected with 6 ng of MO1 or 4 ng of MO2 exhibited upregulation of HuC/D expression (67%, N = 48; Fig. 5B and 5E; Fig. S3). The phenotype appeared more severe when injected with higher dosage of MO1 (12 ng; 77%, N = 52) or MO2 (8 ng; 84%, N = 51; Fig. 5C and 5F). To examine whether the increased HuC/D expression was due to cell number changes or increased expression in individual cells, a z-stack of fluorescence images was acquired by confocal microscopy and the HuC/D positive-cells were counted. The results showed that the number of HuC/D-positive neurons was increased by approximately 2-fold and 4-fold in low dose and high dose morpholino injected embryos, respectively. Western blot analysis also showed a 2.3 fold increase of HuC/D expression in Her8a morphants (Fig. 5J). Co-injection with *her8a* mRNA attenuated the effect of morpholino (8 ng; 78%, N =  50; Fig. 5I) indicating that the neuronal defects in MO1 and MO2 morphants were the result of specific inhibition of Her8a function.

**Figure 6:**
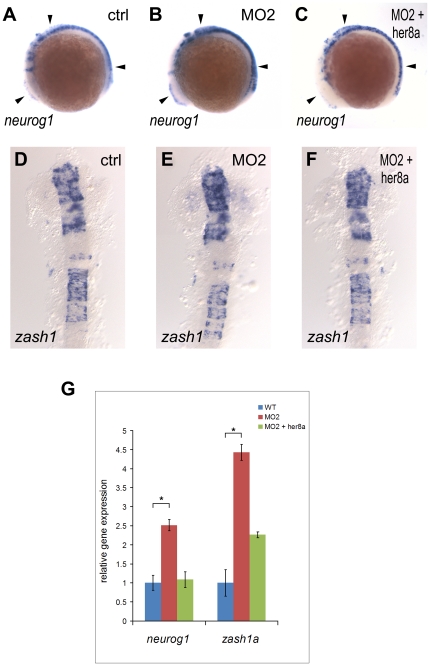
Increased neuronal precursors in Her8a knockdown embryos. Proneural genes were upregulated in the morpholino injected embryos and this effect was rescued by co-injection with *her8a* mRNA. Proneuronal markers *neurogenin1* (A–C) and *zash1* (D–F) were analyzed by *in situ* hybridization. Embryos were injected with control morpholino (A and D), 8 ng of MO2 (B and E), or co-injection of 8 ng of MO2 with 480 pg *her8a* mRNA (C and F). The most dramatic phenotypes are indicated by arrows. Stages of embryos are 6-somite stage (A-C) and 16-somite stage (D–F). (G) qPCR analysis showing the levels of *neurogenin1* and *zash1* are both significantly increased in embryos injected with *her8a* morpholino. * p<0.05.

The increased number of neurons resulting from Her8a knockdown resembles the effect observed in Notch deficient embryos which have been shown to be the result of lost of lateral inhibition and consequently caused premature differentiation of neurons [30]. Since neuronal differentiation is initiated by proneural genes, we next examined whether knockdown of Her8a also affect the expression of proneural markers, *neurogenin1* and *zash1*. The result showed proneural genes were upregulated in the morpholino injected embryos (*neurogenin1*: 74%, N = 68; *zash1a*: 71%, N = 56), and the phenotype could be rescued by *her8a* mRNA injection (*neurogenin1*: 80%, N = 56; *zash1a*: 95%, N = 40) (Fig. 6A–F). Quantitative real time PCR (qPCR) analysis also showed a 2.5 fold and 4.4 fold increase of *neurogenin1* and *zash1a* expression in Her8a morphants, respectively (Fig. 6G). On the contrary, widespread over-expression of *her8a* mRNA alone did not alter the expression of proneural and pan-neuronal markers (Fig. S4), indicating that Her8a alone was not sufficient to inhibit neurogenesis. Subsequently, we analyzed the effect of Her8a in neural stem cells. *Sox2* is expressed in neural stem cells where it maintains the stemness identity and inhibits neurogenesis [31]. We found that *sox2* expression remained intact after Her8a knock down (Fig. S5). To confirm, we used *sox3* as another neural progenitor maker ([32]; [33]) and found the expression of sox3 also remained intact in Her8a morphants (Fig. S5C and S5D). Since Notch signaling-initiated lateral inhibition comes into play after the Sox2/3-positive neural progenitor regions is determined, these data are consistent with the expectation that these stem cell markers would not be altered in Her8a deficient embryos. Taken together, these results suggest that Her8a is required for inhibition of neurogenesis, and loss of Her8a results in premature neural differentiation.

**Figure 7:**
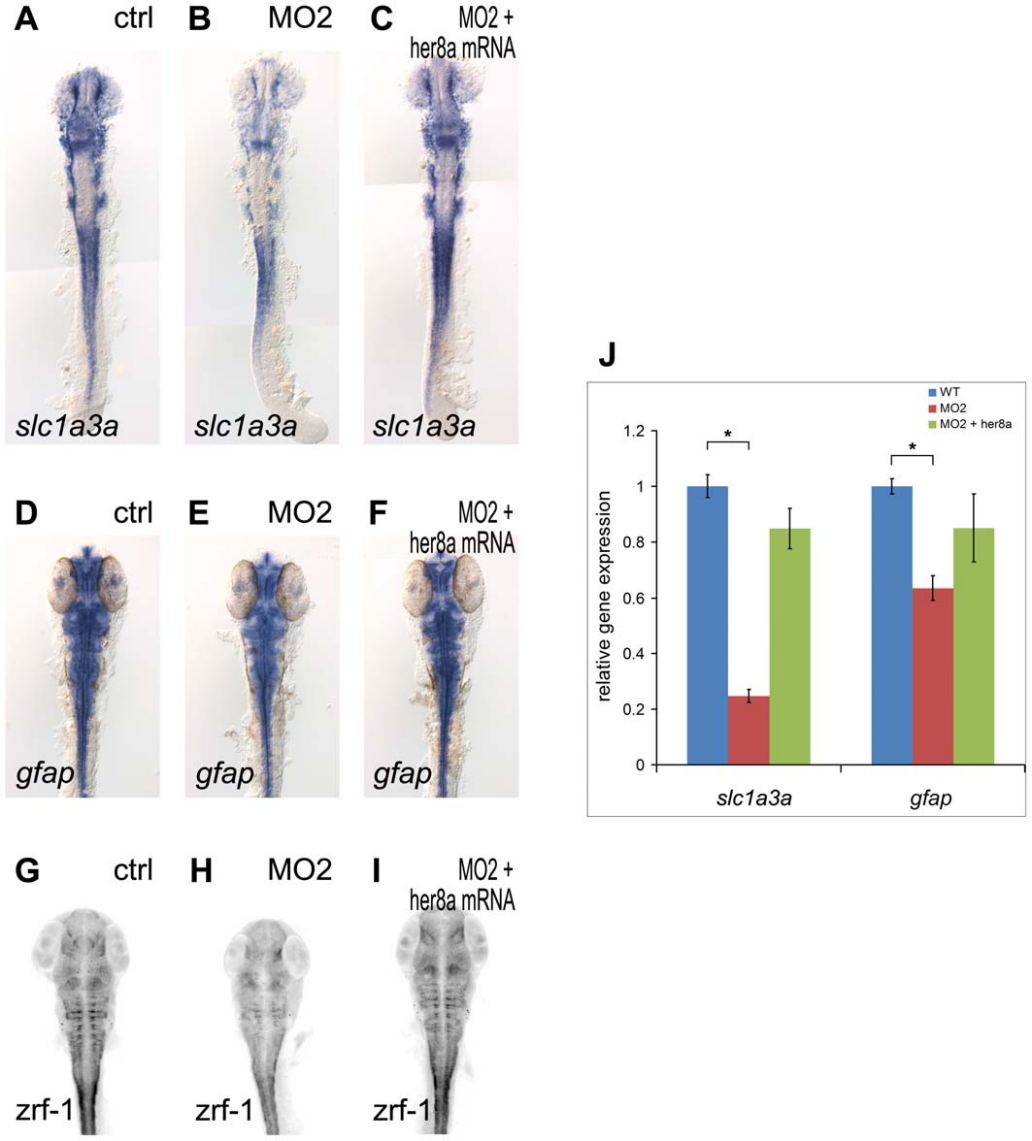
Glial precursors and mature glial cells were reduced in Her8a knockdown embryos. Her8a morpholino injection downregulates the expression of the glial precursor marker *slc1a3a* (A, B) and radial glial markers (*gfap* for radial glial cell body (D, E) and zrf-1 for glial fibers (G, H)), and this effect was rescued by co-injection with *her8a* mRNA (C, F, I). (A, D, G) Control morpholino, (B, E, H) 8 ng of MO2, (C, F, I) Co-injection of 8 ng of MO2 with 0.5 ng *her8a* mRNA. Embryos were harvested at the 18-somite stage (A–C) and 36 hpf (D–I). (A–F) *in situ* hybridization; (G–I) immunohistochemistry with zrf-1 antibody, fluorescent signals were inverted to negative film for a better presentation. (J) qPCR analysis showing the levels of *slc1a3a* and *zash1* and zrf-1 are significantly decreased in embryos injected with *her8a* morpholino. * p<0.05.

Previous studies have shown that H/E(spl) transcription factors not only repress the expression of proneural genes during neurogenesis, but also promote the differentiation of many glial subtypes (reviewed in [1]). In contrast, mouse HES6 inhibits astroglia differentiation *in vitro* [20]. Consistent with this, we found that the early glial marker, *slc1a3a* (*Glast* in mammals, [34], [35]) was reduced in Her8a knockdown embryos (83%, N = 12, Fig. 7A–C, J), suggesting that Her8a is required for gliogenesis. To further confirm the reduction of *slc1a3a* in Her8a morphants was not due to increased glial differentiation, we analyzed the expression of mature radial glial cell markers (zrf-1 antibody for radial glial fibers and *gfap* riboprobe for radial glial cell body) and found the expression were downregulated in Her8a knockdown embryos (Fig. 7 D–J)), supporting our notion that Her8a is required for gliogensis. However, the possibility that the reduction of glial markers was due the bias of neuronal differentiation cannot be ruled out.

Previous studies have shown that morpholinos can cause off-target apoptosis mediated by p53 activation [36]. To rule out the possibility that the effect of *her8a* morpholinos was mediated by off-targeted apoptosis, all *her8a* MOs were co-injected with a *p53* MO and we did not observe any detectable deviation in Her8a morphants with or without *p53* MO (Fig. S6). Since the phenotypes caused by *her8a* morpholino injection could also be rescued by concomitant injection of *her8a* mRNA as shown above, we suggest that the phenotypes in Her8a morphants were the result of specific inhibition of Her8a function independent of p53 activity.

## Discussion

H/E(spl) family members efficiently inhibit neurogenesis by forming homodimers or heterodimers with other H/E(spl) factors. These then bind to target sequences to actively repress the transcription of target genes, or form non-DNA binding heterodimers with bHLH activators such as E47 and inhibit transcriptional activation [3]. We have isolated *her8a*, a member of the E(spl) family in zebrafish. Intriguingly, although we found that knockdown of Her8a resulted in premature neurogenesis and this could be rescued by concomitant over-expression of *Her8a* mRNA, over-expressing Her8a alone failed to efficiently inhibit the formation of post-mitotic neurons. This suggests that Her8a may need to form a heterodimer with other H/E(spl) factors to inhibit neuronal differentiation. In support of this, we did find several Her homologues could interact with Her8a by yeast two-hybrid analysis (data not shown), but whether they can operate together *in vivo* to inhibit neurogenesis remains to be tested.

According to the sequence similarity and conserved intron/exon structure *her8a* is a member of the HES6 subfamily. Strikingly, we found that *her8a* was activated by Su(H)-dependent Notch signaling, in contrast to chick HES6-2 which has been shown to be repressed by Notch signaling [18] and *Xenopus hes6.1* which has been shown not to respond to Notch activation [6] in the developing nervous system. However, it is noteworthy that although *her8a* was significantly downregulated in *mib^ta52b^* embryos suggesting that *her8a* is activated by Notch signaling, residual expression of *her8a* was maintained at the midbrain and the hindbrain rhombomere boundaries in Notch deficient mutants, suggesting that *her8a* may also response to other singling pathways in these regions. In support of this, a previous study in chick embryos showed that *H/E(spl)* genes can respond to FGF and TGFβ signaling at the rhombomere boundaries [37], and other work showed that *H/E(spl)* could be regulated by BMP, TGFβ, JAK-STAT and Ras signaling cascades in several tissue systems (review in [25]). Therefore, *her8a* is positively-regulated by Notch signaling to repress neuronal differentiation, but it may also respond to other signaling pathways in the midbrain and the rhombomere boundaries where the role of Her8a is currently not clear and needs to be further analyzed.

The roles of the H/E(spl) family members are generally evolutionarily conserved in the developing nervous system where they have been demonstrated to repress neurogenesis. In contrast, The Hes6 subfamily have a role distinct from other H/E(spl) members, which is to promote instead of to inhibit neurogenesis. And all Hes6 homologues characterized to date showed a similar function. For example, chicken HES6-2 represses HES5 and cooperates with proneural genes to promote neuronal differentiation [18], mouse HES6 promotes neuronal cell differentiation by suppressing HES1 [13], and *Xenopus* hes6.1 also promotes neuronal differentiation [6]. Interestingly, we found that zebrafish Her8a is essential for the inhibition of neurogenesis, the converse of the earlier demonstrated roles for Hes6 homologues. Previously, another Hes6 homologue named *her13.2* was identified in zebrafish that is required for somite segmentation, but its role in the developing nervous system has not been analyzed [38]. The sequences of two other predicted genes, named *her8.2* and *her13.1*, have been described in zebrafish that also show high similarity to mammalian *Hes6*, but their roles have not yet been studied [7]. Therefore, it is possible that at least one of the above genes could share the functional role of the mammalian HES6 protein to promote neurogenesis. Taken together, our results suggest *her8a* was created by duplication events which allowed functional diversity to develop within the paralogues. However, within this group Her8a is essential to repress neuronal differentiation. Since *her8a* expression is regulated by Notch signaling, these data provide further understanding of how Notch signaling and downstream genes mediate neural cell fate determination.

## Materials and Methods

### Ethics Statement

All experiments were performed in strict accordance to standard guidelines for zebrafish work and approved by the Institutional Animal Care and Use Committee of Chang Gung University (IACUC approval number: CGU04-57 and CGU08-86).

### Sequence comparisons and phylogeny

Amino acid sequences were aligned and displayed using the Vector NTI (Invitrogen). Phylogenetic tree calculation was performed with ClustalX [39]. The GenBank accession numbers of the compared proteins are as follows: zebrafish Her1 (NM_131078), Her2 (NM_131089), Her3 (NM_131080), Her4.1 (NM_001161408), Her4.2 (NM_131090), Her5 (NM_131077), Her6 (NM_131079), Her7 (NM_131609), Her8a (NM_199624), Her8.2 (NM_001166166), Her9 (NM_131873), Her11 (NM_001003886), Her12 (NM_205619), Her13.1 (NM_001017901), Her13.2 (NM_194400), Her15.1 (NM_182875); chicken Hes1 isoform 1 (c-hairy1A, NM_001005848), Hes1 isoform 2 (c-hairy1B, NM_204472), Hes5 (NM_001012695), Hes6-2 (XP_422641); *Xenopus laevis* hes1-a (NM_001087927), hes1-b (NM_001085917), hes2 (NM_001122882), hes3.1 (NM_001088503.1), hes3.3 (NM_001088694), hes4-a (NM_001089105), hes4-b (NM_001088692), hes5.1 (NM_001095627), hes5.2-a (NM_001088505), hes5.2-b (NM_001095626), hes6.1 (BC130161), hes7.1 (NM_001088706), hes7.2 (AAD42783), hes9.1-a (NM_001088237), hes9.1-b (NM_001095628.1); mouse HES1 (NM_008235.2), HES2 (NM_008236.4), HES3 (NM_008237), HES5 (NM_010419.4), HES6 (NM_019479.3), HES7 (NM_033041); human HES1 (NM_005524), HES2 (NM_019089), HES3 (NM_001024598), HES4 (NM_001142467), HES5 (NM_001010926), HES6 (NM_018645), HES7 (NM_001165967).

### Fish Maintenance and Mutants

*Tü* (wild type) and *mib^ta52b^* mutant zebrafish embryos were purchased from the Zebrafish International Resource Center (ZIRC, Oregon, USA) and were raised, maintained and paired under standard conditions. The embryos were staged according to the number of somites, hours post fertilization and days post fertilization [40].

### Constructs Generation

The open reading frame of *her8a* was PCR amplified with the primers 5′-GAATTCGCCACCATGACGGCCTCCAACATGGGC-3′and 5′-GGAATTCCTCACCAGGGCCTCCACATG-3′, which introduce *EcoR*I restriction sites suitable for cloning. The PCR product was digested with *EcoR*I and cloned into the pCS2+ MT vector. PCR amplifications were performed with the high fidelity Pfu polymerase (Promega) and constructs were sequenced to check for the absence of mutations.

### RT-PCR

Total RNA was extracted from zebrafish embryos using standard protocol (TRIzol; Invitrogen) and resuspended in nuclease-free water. The concentration and purity of RNA were measured with a spectrophotometer (NanoDrop Technologies), and contaminating genomic DNA was removed using DNase I (Ambion). Reverse transcription was performed using the Thermoscript RT-PCR system (Invitrogen) priming with random hexamers. Synthesized cDNA was used with the primers (forward, 5′- AGAATTCATGACGGCCTCCAACATGGGC -3′; reverse, 5′- GGAATTCCTCACCAGGGCCTCCACATG -3′) spanning the MO2 binding sequence in standard PCR conditions.

### Histological Analysis

Digoxigenin-UTP labeled riboprobes to detect *her8a*, *her3*, *her4*, *her5*, *her6*, *notch1a*, *notch1b*, *notch2*, *notch3*, *deltaA*, *neurog1*, *zash1*, *sox2*, *sox3*, and *slc1a3a* transcripts were synthesized according to manufacturer's instructions (Roche), and *in situ* hybridizations were performed as described previously [30]. The color reaction was carried out using NBT/BCIP substrate (Roche). For immunohistochemistry, the embryos were blocked in 5% goat serum and anti-HuC/HuD neuronal protein monoclonal 16A11 antibody (1/500 dilution, Invitrogen) or zrf-1 radial glial fibers antibody (1/500 dilution, Zebrafish International Resource Center) was applied. Fluorochrome conjugated antibodies Alexa Fluor 488 (or 594) goat anti-mouse IgG (Invitrogen) was used to detect the primary antibodies. Embryos were mounted with Vectashield mouting medium with DAPI (Vector Laboratories, Inc.). Confocal Microscopy was performed using a Zeiss LSM 510 microscope.

### RNA and Morpholino Injection

Capped RNA encoding the full coding sequence of Her8a, constitutive-active Su(H), and dominant-negative Su(H) [29] was prepared as described previously [41]. The *Su(H)* constructs were kindly provided by Chris Kintner. Antisense morpholino oligonucleotides were purchased from Gene Tools, LLC (Oregon, USA). Two morpholinos against *her8a* were used: MO1 (CTTATTACTGCCGGAGGTTGTACCC) that overlaps the ATG start codon, and MO2 (TATTAAACTTAAGGGTGTCGTTAGA) that corresponds to intron2-exon3 boundary region sequence. A *p53* morpholino with the sequence GACCTCCTCTCCACTAAACTACGAT (Gene Tools, LLC) was used to rule out the possibility that the effect of *her8a* MOs was mediated through an off-targeted p53 activation. All injections were performed at the one to two-cell stage and mRNAs or morpholinos were introduced into blastomeres.

### Quantitative analysis

For quantitative real time PCR (qPCR), embryos were homogenized in TRIzol reagent (Invitrogen) and total RNA was extracted using a standard method. cDNA was synthesized from total RNA with random hexamer priming using RevertAid First Strand cDNA Synthesis Kit (Fermentas). qPCR was performed on an ABI StepOne™ Real-Time PCR System (Applied Biosystems) with SYBR green fluorescent label (Fermentas). Primers for *ngn1* (F: 5′-CGCACACGGATGATGAAGACTCGCG-3′; R: 5′-CGGTTCTTCTTCACGACGTGCACAGTGG-3′), *zash1* (F:5′-ACCGGGTGAAGCTTGTGAAC-3′; R: 5′-CGTCATGCYCGTCCAGAAGTT-3′), *slc1a3* (F: 5′-GTAACGGGGAGACGCGTCTGCAGCG-3′; R: 5′-GATTATTCCCACGATGACGGCGGCG-3′), *gfap*(F: 5′-ACTGAGGAGTGGTATCGCTCAAA-3′; R: 5′-AGACCCACGGAGAGATTCCA-3′)and *gapdh* (F: 5′-ACCCGTGCTGCTTTCTTGAC-3′; R: 5′-GACCAGTTTGCCGCCTTCT-3′) were used. Gene expression levels were normalized to *gapdh* and assessed using the comparative C_T_ (40 cycles) according to the manufacturer's instructions (Applied Biosystems).

For Western blot analysis, embryos were homogenized in SDS lysis buffer. 60ug were loaded on 12% SDS polyacrylamide gel and transferred to a PVDF membrane and detected with anti-HuC/HuD monoclonal antibody (1∶1000, Invitrogen). After washes, membranes were incubated with goat anti-Mouse HRP-conjugated secondary Ab (Chemicon) and developed with ECL(Millipore). Band intensities were quantified using Multi Gaugre analysis software.

Statistical analysis was performed by student's *t*-test using Microsoft Excel® 2007. The significance level was set at P<0.05. All Reaction was performed in triplicate for each sample.

## Supporting Information

Figure S1 **Expression comparison of** ***notch*** **homologues,** ***her3*** **,** ***her4*** **and** ***neurogenin1*** **.** Expression of *notch* homologues and known Notch target, *her3* and *her4*, and *neurogenenin1* analyzed by *in situ* hybridization. Name of the gene analyzed shown on the top row and stages of embryos shown on the left column. Embryos were flat-mounted, dorsal view.
(TIF)

Figure S2 **Embryos injected with MO1 or MO2 resulted in very similar morphological phenotypes.** (A, B) Embryos injected with control morpholino analyzed at 5 days post fertilization. (C, D) Injection of 6 ng of MO1 or 4 ng of MO2 (lower dosage) caused very similar phenotype exhibiting pericardial edema. (E, F) Embryos injected with 12 ng MO1 or 8 ng of MO2 (higher dosage) show an identical phenotype including brain malformation and edemas in eyes, pericardial sac and the abdominal cavity.
(TIF)

Figure S3 **Embryos injected with MO1 exhibit upregulation of HuC/D expression.** (A, B) Embryos injected with control morpholino. (C, D) Injection of 6 ng of MO1 (low dose) resulted in upregulation of HuC/D expression (E, F) 12 ng of MO1 injection (high dose) displayed more dramatic upregulation of HuC/D.
(TIF)

Figure S4 **Over-expression of** ***her8a*** **mRNA did not alter the expression of proneural and pan-neuronal markers.** Embryos were analyzed by immunohistochemistry with HuC/D antibody (A, B) and *in situ* hybridization with *neurogenin1* (C, D) or *zash1* (E, F). (A, C, E) Embryos were injected with *GFP* mRNA as control. (B, D, F) Injection of *her8a* mRNA revealed no significant deviation.
(TIF)

Figure S5 **No significant alteration can be detected in** ***sox2*** **or** ***Sox3*** **expressing neural progenitors in Her8a morphants.** Embryo injection with control morpholino (A and C) or 8 ng of MO2 (B and D) analyzed with *sox2* (A and B) or *sox3* (C and D) riboprobes. *sox2* and *sox3* were expressed in the neural precursors located within the neuroectodermal region (arrowheads). 75% epiboly; dorsal view, animal pole toward the top.
(TIF)

Figure S6 **The phenotypes in Her8a morphants were not caused by none specific p53 activation.** Embryos co-injected *her8a* MOs with *p53* MO (C, F, I, L, O, R) were compared to Her8a morphants (B, E, H, K, N, Q) and did not cause any detectable deviation analyzed with all markers tested. (A, D, G, J, M, P) Embryos injected with control morpholino.
(TIF)
