# Supplementary figures and images for: Zebrafish Her8a Is Activated by Su(H)-Dependent Notch Signaling and Is Essential for the Inhibition of Neurogenesis
Source: PLoS One. 2011 Apr 26;6(4):e19394. doi: 10.1371/journal.pone.0019394 (PMC3082574; doi:10.1371/journal.pone.0019394)

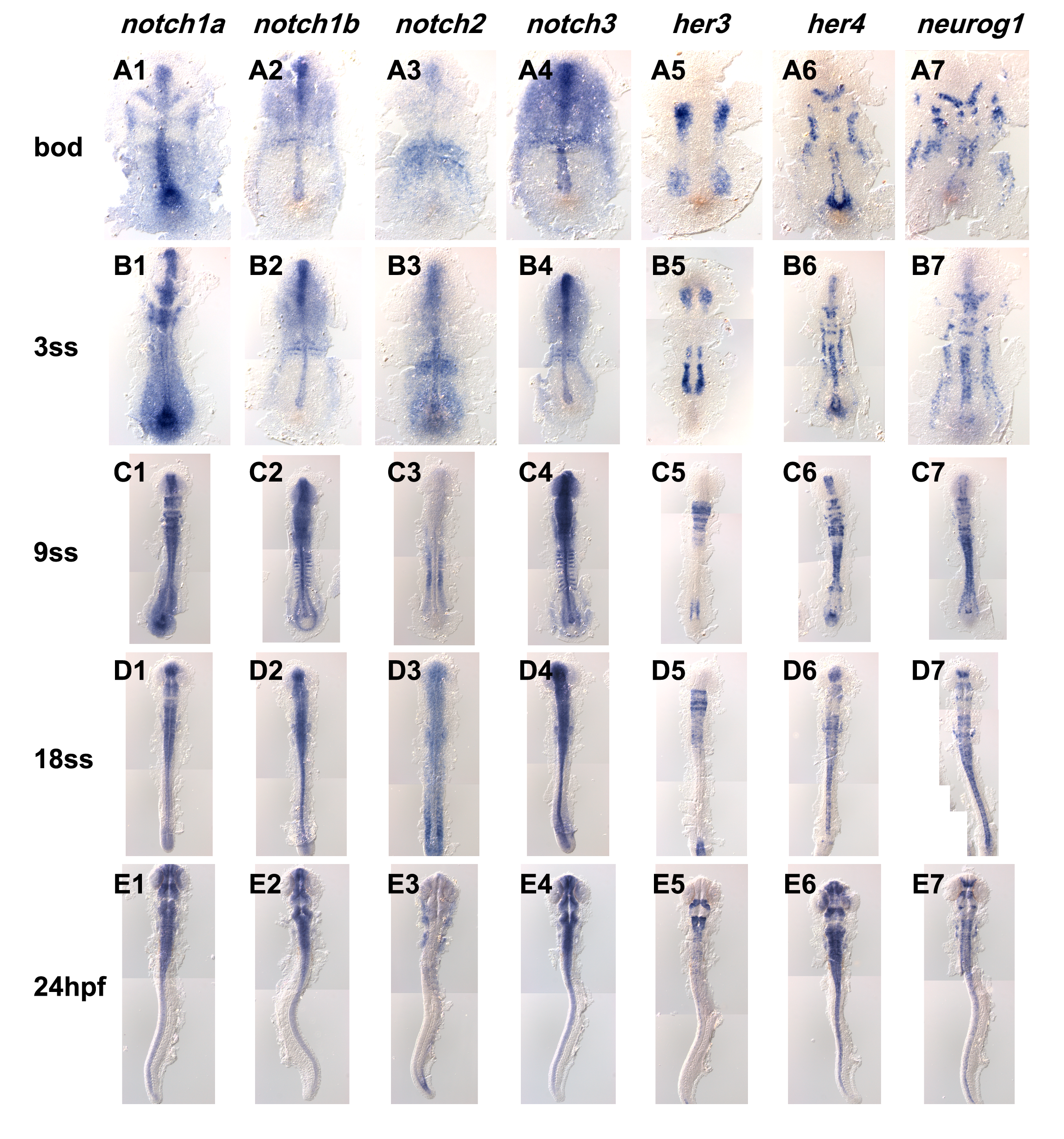

Supplement: Figure S1 — Expression comparison of notch homologues, her3 , her4 and neurogenin1 . Expression of notch homologues and known Notch target, her3 and her4, and neurogenenin1 analyzed by in situ hybridization. Name of the gene analyzed shown on the top row and stages of embryos shown on the left column. Embryos were flat-mounted, dorsal view. (TIF) [file pone.0019394.s001.tif]

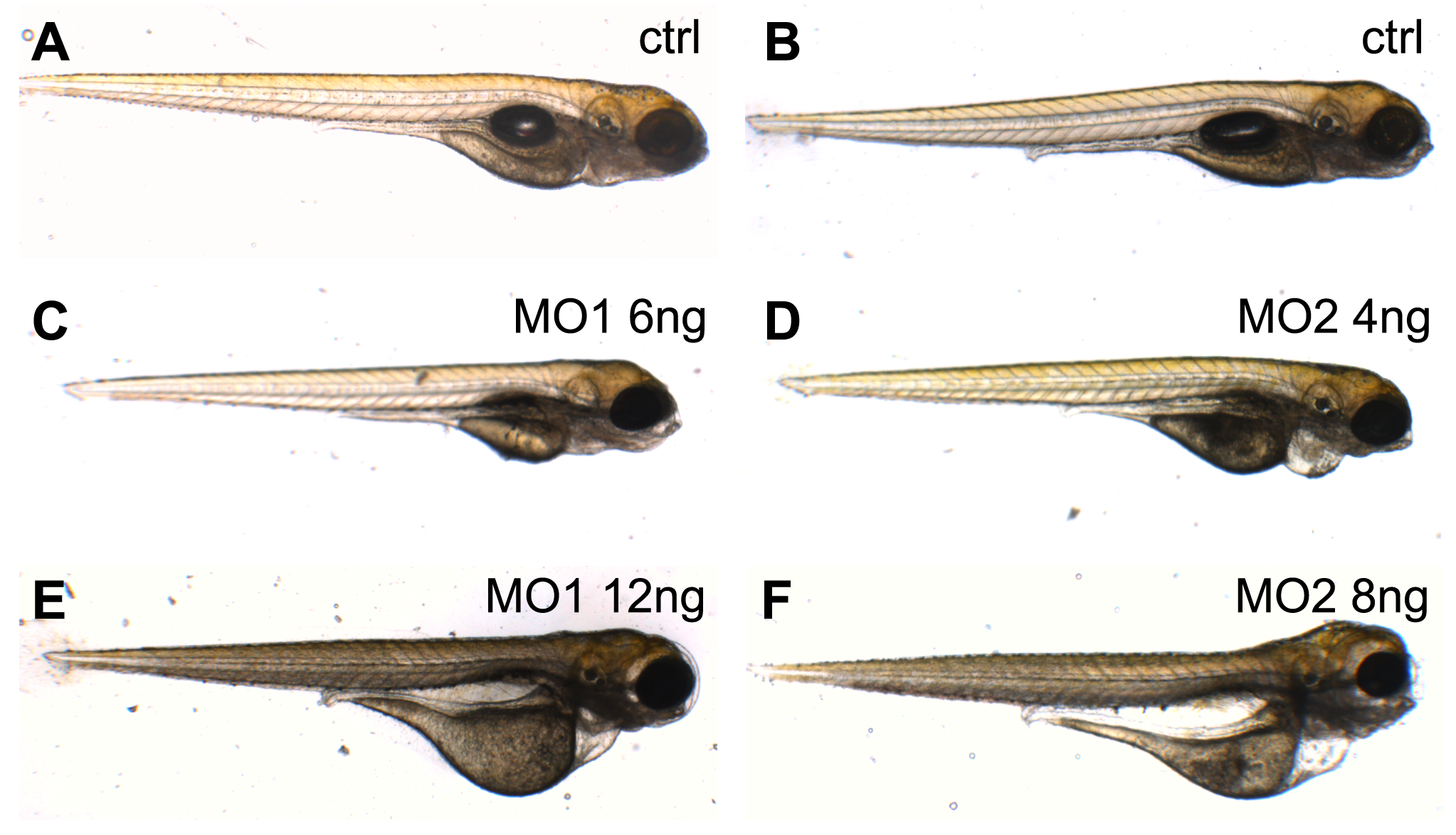

Supplement: Figure S2 — Embryos injected with MO1 or MO2 resulted in very similar morphological phenotypes. (A, B) Embryos injected with control morpholino analyzed at 5 days post fertilization. (C, D) Injection of 6 ng of MO1 or 4 ng of MO2 (lower dosage) caused very similar phenotype exhibiting pericardial edema. (E, F) Embryos injected with 12 ng MO1 or 8 ng of MO2 (higher dosage) show an identical phenotype including brain malformation and edemas in eyes, pericardial sac and the abdominal cavity. (TIF) [file pone.0019394.s002.tif]

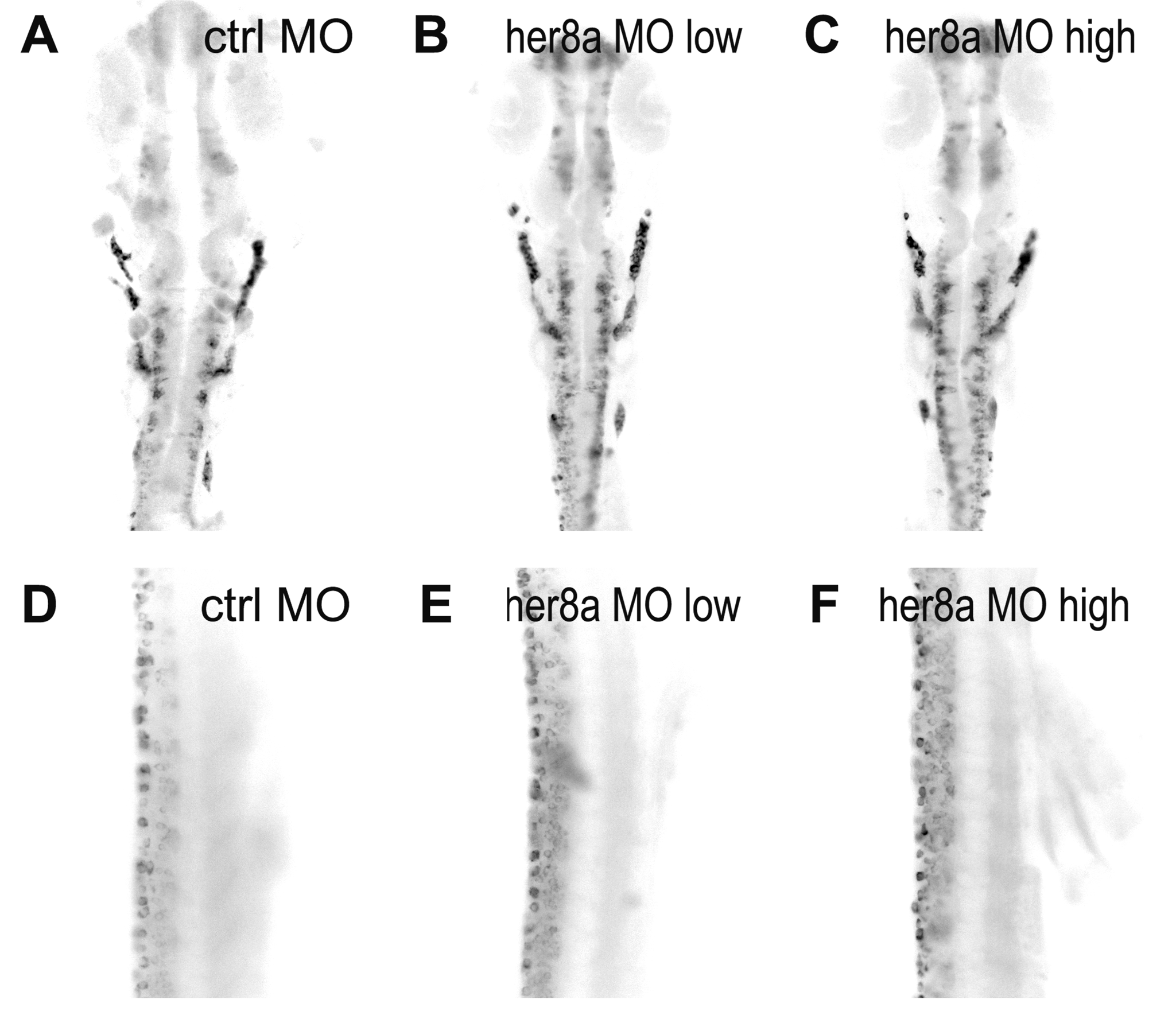

Supplement: Figure S3 — Embryos injected with MO1 exhibit upregulation of HuC/D expression. (A, B) Embryos injected with control morpholino. (C, D) Injection of 6 ng of MO1 (low dose) resulted in upregulation of HuC/D expression (E, F) 12 ng of MO1 injection (high dose) displayed more dramatic upregulation of HuC/D. (TIF) [file pone.0019394.s003.tif]

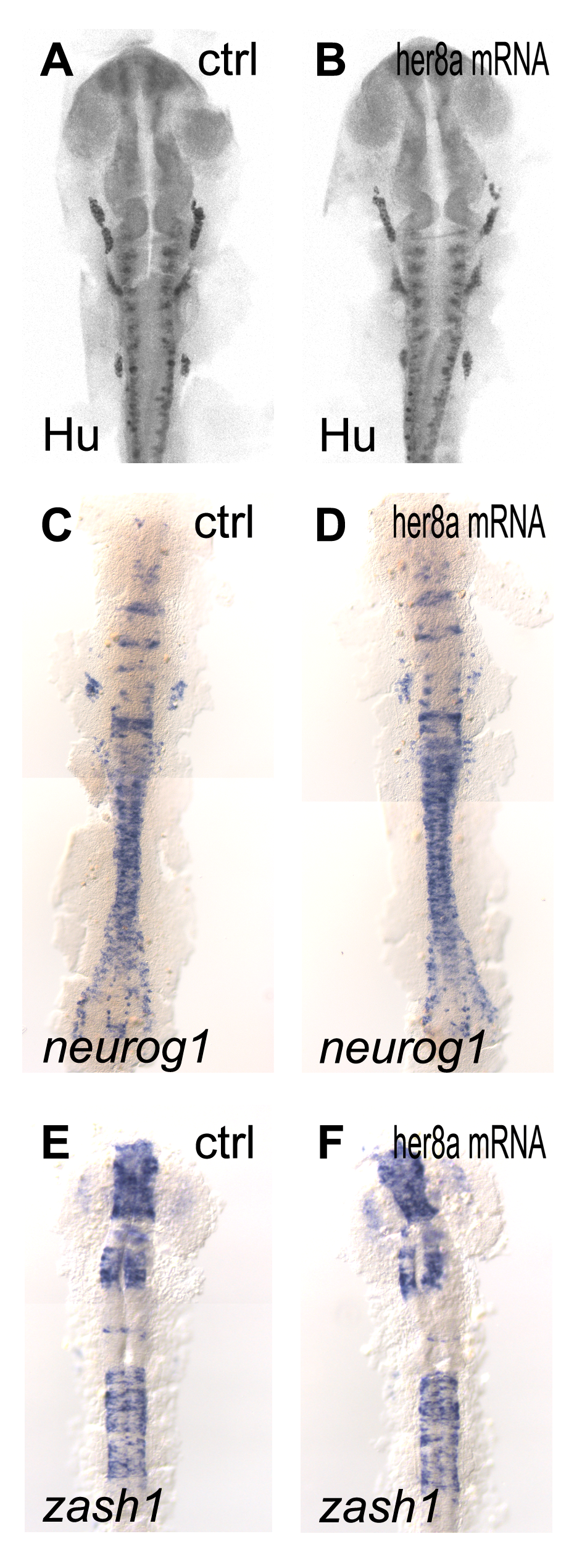

Supplement: Figure S4 — Over-expression of her8a mRNA did not alter the expression of proneural and pan-neuronal markers. Embryos were analyzed by immunohistochemistry with HuC/D antibody (A, B) and in situ hybridization with neurogenin1 (C, D) or zash1 (E, F). (A, C, E) Embryos were injected with GFP mRNA as control. (B, D, F) Injection of her8a mRNA revealed no significant deviation. (TIF) [file pone.0019394.s004.tif]

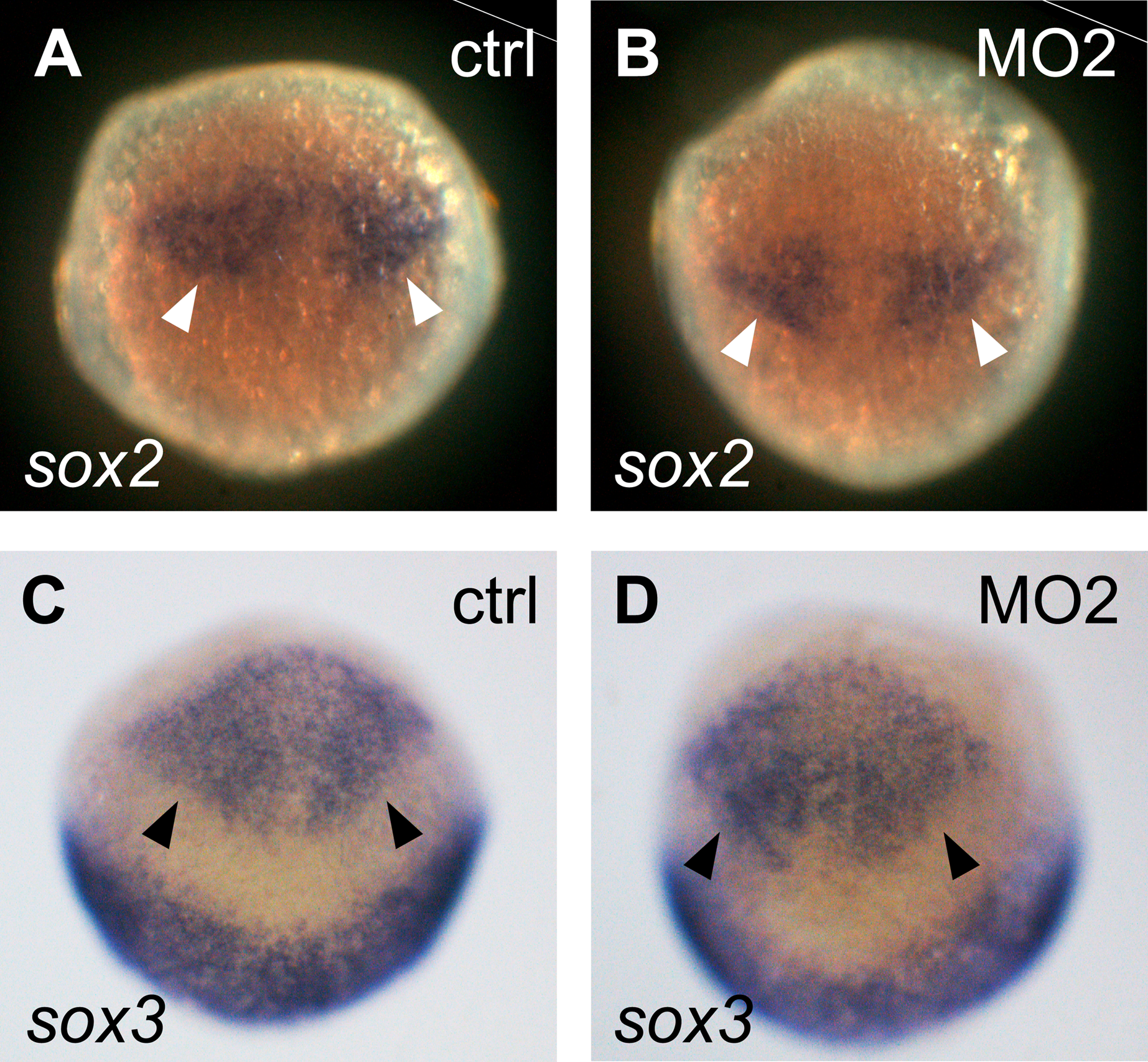

Supplement: Figure S5 — No significant alteration can be detected in sox2 or Sox3 expressing neural progenitors in Her8a morphants. Embryo injection with control morpholino (A and C) or 8 ng of MO2 (B and D) analyzed with sox2 (A and B) or sox3 (C and D) riboprobes. sox2 and sox3 were expressed in the neural precursors located within the neuroectodermal region (arrowheads). 75% epiboly; dorsal view, animal pole toward the top. (TIF) [file pone.0019394.s005.tif]

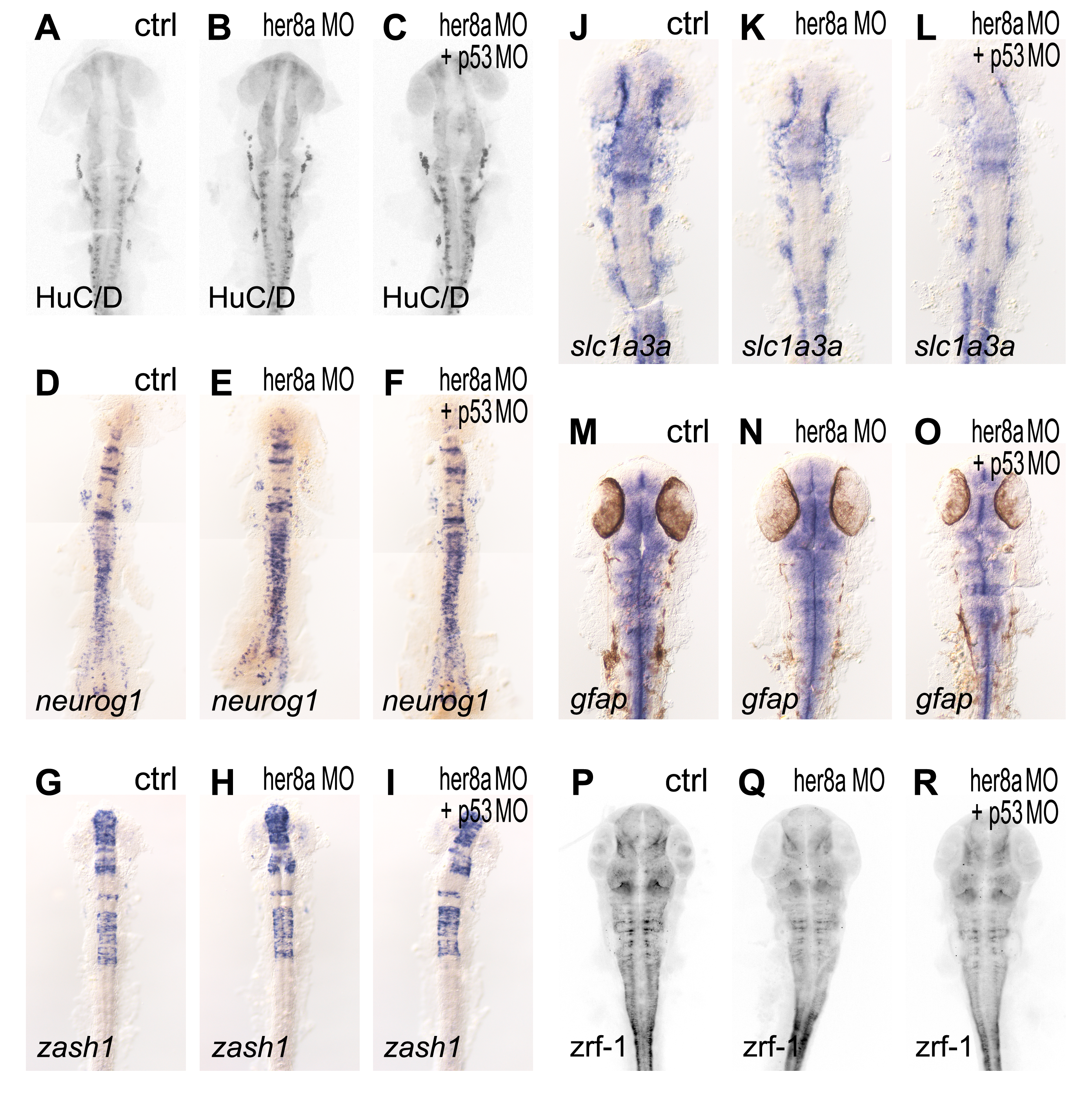

Supplement: Figure S6 — The phenotypes in Her8a morphants were not caused by none specific p53 activation. Embryos co-injected her8a MOs with p53 MO (C, F, I, L, O, R) were compared to Her8a morphants (B, E, H, K, N, Q) and did not cause any detectable deviation analyzed with all markers tested. (A, D, G, J, M, P) Embryos injected with control morpholino. (TIF) [file pone.0019394.s006.tif]
